# Effect of Yam Flour Modified with Plasma-Activated Water Combined with Extrusion Treatment on the Quality of Chinese Noodles

**DOI:** 10.3390/foods14010077

**Published:** 2024-12-31

**Authors:** Miaomiao Shi, Yirui Chen, Xiaopei Zhu, Xiaolong Ji, Yizhe Yan

**Affiliations:** 1 College of Food and Bioengineering, Zhengzhou University of Light Industry, Zhengzhou 450001, China; chengzi3090@126.com (M.S.); cheniiu09@126.com (Y.C.); zxiaopei0217@163.com (X.Z.); yanyizhe@zzuli.edu.cn (Y.Y.); 2 National & Local Joint Engineering Research Center of Cereal-Based Foods (Henan), Zhengzhou 450001, China

**Keywords:** modified yam flour, yam noodles, textural property, in vitro digestive properties

## Abstract

Yam noodles were produced by replacing high-gluten wheat flour with yam flour modified with plasma-activated water and twin-screw extrusion (PAW-TSE). The effects of varying amounts of modified yam flour on the color, cooking characteristics, texture, and in vitro digestibility of the noodles were investigated. As the amount of modified yam flour increased, the noodles became darker in color, while the bound water content increased, and the free water content decreased. The modified yam flour also affected the cooking properties, reducing the optimal cooking time, decreasing the water absorption, and increasing the cooking loss. Textural analysis revealed that the addition of modified yam flour improved the texture of raw noodles, enhancing their elasticity and chewiness after cooking, thus providing a better eating experience. Furthermore, the modified yam flour increased the resistant starch content, thereby enhancing the nutritional value of the noodles. These findings provide valuable insights for food manufacturers seeking to develop healthier and more appealing noodle products, potentially leading to greater consumer acceptance and market success.

## 1. Introduction

Noodle products are traditional staple foods with a long history in China. Noodles, rice, and steamed buns remain the three main staples in people’s diets. Noodles are a traditional food in many Asian countries, known for their high nutritional value, ease of preparation, and delicious flavor. In recent years, noodles have also gained increasing popularity in many Western countries [1]. With the continuous development of the economy and growing consumer awareness of healthy lifestyles, there has been a greater focus on noodles that incorporate natural proteins and other functional ingredients of plant origin [2]. However, most noodles on the market are currently made primarily from wheat flour, resulting in a relatively simple nutritional profile. In light of this, the food industry has begun to focus more on the development of noodles with unique nutritional characteristics [3].

Dietary fiber can slow down the digestion of food and help control the rapid increase in postprandial blood glucose levels [4]. Yam (*Dioscorea opposita* Thunb.) is a nutritious ingredient that has been widely used in the food industry due to its high dietary fiber content, low glycemic index (GI), and other beneficial properties. In China, yam is considered an economically valuable food with significant development potential and future market prospects, and it is classified as a medicinal food product [5]. One of its main derivatives, yam noodles, not only offer good taste and nutritional value but also meet the needs of diverse consumers. However, there are certain drawbacks in the traditional processing methods for yam noodles, such as poor elasticity and chewiness, which result in noodles that are prone to breaking [6,7]. To address these issues, researchers have explored various physical and chemical modification methods in recent years, including plasma-activated water (PAW) treatment and twin-screw extrusion (TSE) treatment.

The liquid obtained by treating distilled water with plasma is called plasma-activated water (PAW). Plasma, as the fourth fundamental state of matter, is an ionized gas primarily composed of photons, ions, free electrons, and neutral or excited-state atoms with a net neutral charge [8]. During the plasma discharge process, many reactive oxygen and nitrogen species are generated in the ionized gas, and they interact with the water molecules in distilled water, leading to the production of numerous active chemical substances in PAW [9]. The main active substances in PAW include hydrogen peroxide (H_2_O_2_), nitrate (NO^3−^), nitrite (NO^2−^), ozone (O_3_), hydroxyl radicals (-OH), superoxide (O^2−^), nitric oxide (NO), and peroxynitrite (ONOO-) [10]. In addition to these reactive substances, PAW also undergoes significant changes in its physicochemical properties compared to distilled water, such as water acidification, a decrease in pH, and an increase in redox potential and conductivity. These active substances can interact with components in food, thereby altering its structure and properties. The use of PAW is not affected by the irregular shape of the treated raw materials, and it can effectively improve the uniformity of treatment results [11]. PAW, due to its rich content of various highly reactive oxygen and nitrogen species, is capable of forming an acidic environment while also possessing the fluidity and permeability of water. It has found widespread applications in different areas of the food industry. PAW can be used for the preservation and color protection of meat products. Common color protectants in traditional meat products include nitrates and nitrites, but these substances raise public concerns because they can produce carcinogenic compounds during their reactions. Due to the abundant nitrite and nitrate ions in PAW, Jung et al. [12] first proposed using PAW as a substitute for nitrites in meat products and demonstrated that PAW has color-preserving effects on fresh minced meat without promoting lipid oxidation. In addition, PAW has preservative effects on vegetables and fresh-cut fruits. Studies by Guo et al. have shown that PAW soaking treatment can effectively eliminate microorganisms on the surfaces of grapes, strawberries, and button mushrooms, significantly extending the shelf life of these products while having no significant impact on the physicochemical parameters such as color, firmness, pH, and antioxidant capacity of fruits and vegetables [13,14,15]. Furthermore, PAW can reduce pesticide residues in fresh fruits and vegetables. A study by Sarangapani et al. [16] found that PAW achieved a maximum pesticide degradation rate of 86% for chlorpyrifos and 74% for diazinon in grape samples, with a slightly lower degradation rate for strawberries. The degradation of pesticides is attributed to chemical induction via reactive substances such as nitrites, nitrates, and hydrogen peroxide, as well as PAW’s high oxidation potential. Therefore, PAW has great potential for application in the sterilization, preservation, and reduction in pesticide residues in fresh fruits and vegetables. With the widespread application of PAW in the food industry, its use in starch modification has also become a popular topic.

Twin-screw extrusion (TSE) is a food processing technology that offers continuous operation capabilities. The key characteristics of this technology include a high temperature, high pressure, high shear force, and high shock force [17]. Due to its high efficiency and controllability, twin-screw extrusion has been widely used in food processing. In the past decade, twin-screw extrusion technology has achieved significant results in the modification of starches and cereals. A study by Ali et al. [18] indicated that processing temperature affects the structure and functional properties of puffed corn and potato starch. Compared with native starch, the extruded starch showed reduced crystallinity, higher solubility, and improved in vitro digestibility. Wang et al. [19] explored the effects of moisture content on extruded rice starch, finding that extrusion under high moisture conditions enhanced the gelatinization of corn starch and reduced its orderliness. Yan et al. [20] found that thermal-moisture treatment caused a decrease in the crystallinity and resistant starch content of corn starch while increasing its solubility and digestibility. Cheng et al. [21] demonstrated that low-temperature extrusion disrupted the crystalline structure of potato starch, increasing both the water absorption and solubility indexes. Wani et al. [22] showed that high-temperature, short-time extrusion treatment reduced the amylose content of chickpea starch, resulting in decreased viscosity and color properties, but increased its solubility. Therefore, extrusion treatment can effectively improve the structure and physicochemical properties of starch.

Although a few studies have explored the effects of PAW and extrusion treatment on starch modification, further research is needed to investigate the specific impact of different amounts of PAW-TSE treated yam powder on noodle quality [23]. The aim of this study was to evaluate the impact of various amounts of PAW-TSE-treated yam flour on noodle quality by systematically using high-gluten wheat flour noodles as a control. Specifically, the study replaced high-gluten wheat flour with varying amounts of yam flour, examining how the quantity of modified yam flour affected noodle quality, including texture, color, and cooking characteristics. Additionally, in vitro digestion experiments were conducted to analyze the starch digestibility of noodles with modified yam flour. This study aims to provide insights into the deep processing, comprehensive development, and utilization of yam, offering both theoretical foundations and technical support for incorporating modified yam flour into functional noodle products.

## 2. Materials and Methods

### 2.1. Materials

Fresh yam (2000 g, *Dioscorea opposita* Thunb. cv. Tiegun) was purchased from Wenxian in Henan Province, China. The wheat flour was produced by Hebei Jinshahhe Flour Industry Group Co., Ltd., Xingtai, Hebei, China (1000 g of durum wheat was used to produce high-gluten flour). All raw materials used in this experiment were food-grade. Amyloglucosidase A7095 (260 U/mL) and porcine pancreatin P7545 (8 × USP) were obtained from Sigma-Aldrich (St. Louis, MO, USA). Glucose oxidase-peroxidase (GOPOD) and starch assay kits were acquired from Megazyme International Ireland Ltd. (Wicklow, Ireland). All enzymes and chemical reagents used in this experiment were of analytical grade.

### 2.2. Methods

#### 2.2.1. Preparation of Yam Flour

Yam flour was extracted using the method described in our previous study [24]. The fresh yam was cleaned, peeled, and cut into thin slices of 4–5 cm. The slices were washed with deionized water and then drained and soaked in a mixed color-preserving solution consisting of 1% Vitamin C (Vc) and 2% citric acid for 20 min. After color preservation, the slices were drained and placed in an electric hot air drying oven to be dried at 40 °C for 24 h. Once dried, the slices were crushed and sieved through a 100-mesh screen to obtain yam flour.

#### 2.2.2. Preparation of PAW

Plasma-activated water (PAW) was obtained by treating 100 mL of distilled water with an atmospheric pressure plasma jet (APPJ) device (TS-PL200, Easton Geake Automation Equipment Co., Ltd., Shenzhen, China) for 120 s. The discharge distance between the plasma jet probe and the water surface was 25 mm, with an input power of 750 W and an air pressure of 0.18 MPa during operation.

#### 2.2.3. PAW Extrusion Processing Yam Flour

The moisture content of the yam flour was adjusted to 40% by adding plasma-activated water to 50 g of yam flour. The mixture was extruded using a bench-top twin-screw extrusion laboratory machine (Process 11, Thermo Fisher Scientific, Waltham, MA, USA) at an extrusion temperature of 90 °C and a screw speed of 180 r/min. The extrudates were dried in an oven at 45 °C, crushed, ground, and sieved. The resulting sample was recorded as PAW-EYF.

#### 2.2.4. Preparation of Yam Noodles

The produced PAW-EYF was added to high-gluten wheat flour at concentrations of 0%, 5%, 10%, 15%, 20%, and 25% to make noodles. For the subsequent color analysis and cooking properties analysis, the required samples were noodles made from 30 g of wheat flour and yam powder, along with an appropriate amount of water, processed through a series of steps.

The preparation process began with weighing a specific amount of salt, which was then dissolved in warm water for later use. Next, modified yam flour was mixed with high-gluten wheat flour in the specified proportions, and the saltwater solution (containing 40% distilled water and 2% salt by weight of the flour) was added to the flour mixture. This mixture was kneaded for 10 min to form a dough. After kneading, the dough was wrapped in plastic wrap and allowed to rise at room temperature for 20 min. Once the dough had risen, it was rolled multiple times using a pasta press (FKM-150, Junxifu Kitchenware Co., Ltd., Yongkang, Zhejiang, China). The dough sheet was pressed and folded four times at gear setting 1, followed by two times at gear setting 3, two times at gear setting 5, and finally, two times at gear setting 7. This process produced a smooth sheet approximately 1 mm thick. The pressed pasta sheets were then cut into strips measuring 2.5 mm in width, 15 cm in length, and 1 mm in thickness.

The yam noodles prepared with varying PAW-EYF additions were designated as PAW-EYF 0%, PAW-EYF 5%, PAW-EYF 10%, PAW-EYF 15%, PAW-EYF 20%, and PAW-EYF 25%.

#### 2.2.5. Determination of Chromaticity

The above pasta sheets were cut into 4 cm × 4 cm squares. The color was measured using a Color Difference Analyzer (Ci6x, X-Rite, Shanghai, China). The Ci6x color difference meter is configured with a D65 light source and a 10° observation angle. These standard settings ensure measurement accuracy and consistency, making it particularly suitable for evaluating the color of noodles. Prior to testing, background calibration was conducted using white and black. The values for brightness (L*), red–green balance (a*), and yellow–blue balance (b*) were recorded [25]. As the absolute values of these measurements increased, the corresponding color became darker [6]. The whiteness index (WI) of the sample was calculated using the following Formula (1):

(1) $$\mathrm{WI}=100-\sqrt{{(100-L^{*})}^{2}+{a^{*}}^{2}+{b^{*}}^{2}}$$

#### 2.2.6. Determination of Moisture Distribution

We used low-field nuclear magnetic resonance (NMR) technology to analyze the moisture distribution in noodles, thereby investigating the effects of PAW-EYF addition on the moisture content, state, and migration within the noodles. The equipment used is a low-field nuclear magnetic resonance analyzer, manufactured by Shanghai NuoMai Electronics Technology Co., Ltd. (Shanghai, China), with the model number NM-120. The noodles were cut into 3 cm strips and sealed in a specialized MRI test tube. The CPMG pulse sequence was selected for measurement. The measurement parameters were as follows: the number of echoes (C_0_) was 1200, the number of sampling points (TD) was 166,398, the sampling frequency (SW) was 100.00 kHz, the half-echo time (TE) was 0.208 ms, and the number of repetitive scans (NS) was 64.

#### 2.2.7. Determination of Textural Properties

##### Raw Yam Noodles

The method described by Cankurtaran et al. was referenced and slightly modified for the texture determination of yam noodles using a texture instrument (TA-XT plus, Stable Micro System, Godalming, UK) [26]. Fresh yam noodles were cut into short strips measuring 2.5 cm in length. Three strips were then taken and placed horizontally under the probe at intervals of 0.5 cm, compressed twice using TPA mode, and the P/36R model probe was selected. The measurement parameters were as follows: pre-test speed of 3 mm/s, mid-test speed of 1 mm/s, post-test speed of 1 mm/s, strain factor of 50%, and trigger force of 5 g. The results were obtained using the P/36R model probe in TPA mode.

##### Cooked Yam Noodles

The deionized water (500 mL) was poured into a pot and brought to a boil at 100 °C. Twenty yam noodles (each 15 cm long) were added to the boiling water and cooked for the optimal cooking time. After cooking, the noodles were removed from the pot and rinsed under deionized water for 1 min to cool them to room temperature. The rinsed noodles were then drained thoroughly to remove excess water. Finally, the noodles were cut into smaller pieces, and the determination was carried out using the same measurement method as for the raw yam noodles.

#### 2.2.8. Determination of Cooking Characteristics

##### Optimal Steaming Time

The deionized water (500 mL) was measured and poured into a pot; then, it was heated until it boiled. Once the water reached a boil, twenty yam noodles (each 15 cm long) were added to the pot. After 2 min of cooking, the sampling process was initiated by removing one noodle every 5 s. Each noodle was laid flat on a transparent glass plate, and a knife was used to cut through its cross-section. The time at which the white core disappeared from the center of the noodle was recorded, representing the optimal steaming duration.

##### Ratio of Broken Stripes

Twenty yam noodles (each 15 cm long) were selected and placed in 500 mL of boiling water. The noodles were cooked for the optimal steaming time, after which the breaking of the noodles was observed. The number of broken noodles was recorded as *n*. The rate of broken noodles was calculated using the following Formula (2):

Breakage rate (%) = *n*/20 × 100 (2)

##### Water Absorption of Dry Matter

Twenty yam noodles (each 15 cm long) were selected and weighed. The noodles were then placed in 500 mL of boiling water and cooked for the optimal steaming time. After cooking, they were quickly removed, cooled, and rinsed with deionized water for 1 min. The noodles were then placed on filter paper and evenly dispersed for 5 min before being weighed again. The dry matter water absorption rate of the noodles was calculated using the following Formula (3):

Water absorption (%) = [M_2_ − M_1_ × (1 − W)]/[M_1_ × (1 − W)] × 100 (3)

In the formula, M_1_ represents the weight of the yam noodles before cooking, M_2_ represents the weight of the yam noodles after cooking, and W represents the moisture content of the yam noodles before cooking.

##### Cooking Loss in Noodle Preparation

The remaining broth used to measure the water absorption of dry matter was cooled to room temperature and then transferred to a 500 mL volumetric flask for standardization. A 50 mL sample of the broth was measured and poured into a pre-weighed petri dish. The dish was then dried in an oven at 105 °C until a constant weight was achieved. The rate of substance loss was calculated using the following Formula (4):

Loss rate (%) = [10 × (M_4_ − M_3_)]/[M_1_ × (1 − W)] × 100 (4)

In the formula, M_3_ represents the weight of the pre-weighed petri dish, while M_4_ represents the weight of the petri dish after the addition of 50 mL of noodle broth.

#### 2.2.9. Determination of Microstructure

Noodles (3 g) with varying additions of PAW-EYF were selected for vacuum freeze-drying. The freeze-dried noodles were sliced and fixed onto the carrier stage using conductive adhesive at a magnification of 1000 times. The starch samples were attached to the carrier stage with conductive adhesive and then subjected to gold coating for 40 s. The carrier stage was placed under a scanning electron microscope (Regulus 8100, Hitachi Corporation, Chiyoda, Tokyo, Japan) operating at an accelerating voltage of 3 kV for observation at a magnification of 1500 times.

#### 2.2.10. Determination of In Vitro Digestibility of Starch

The in vitro starch digestibility was assessed using the method described in our previous study [27]. The sample (dry basis, 200 mg) was mixed with 4 mL of sodium acetate buffer (pH = 5.2, 0.1 mol/L) into a 50 mL centrifuge tube. The sample was incubated in a water bath thermostat at 100 °C (190 rpm) for 30 min and then cooled to room temperature. A total of 3.0 g of porcine pancreatic enzyme was vortexed with 21 mL of distilled water for 10 min and then centrifuged. Next, 1.755 mL of distilled water and 0.795 mL of amyloglucosidase were mixed with the centrifuged supernatant to form an enzyme mixture. The enzyme mixture (1 mL) was added to each of the cooled centrifuge tubes and hydrolyzed at 37 °C with shaking. At 20 and 120 min, 0.1 mL of the supernatant was taken and inactivated with 4 mL of 70% ethanol before centrifugation. After centrifugation, 0.1 mL of the supernatant was mixed with 3 mL of GOPOD, and the color was developed at 45 °C for 20 min. The absorbance values were measured at 510 nm using a multifunctional microplate reader (Tecan Spark 20M, Diken Laboratory Equipment Co., Ltd., Shanghai, China), with standard glucose solution and deionized water as the standard control and the in vitro blank control, respectively [28]. Simultaneously, 0.1 mL of distilled water and a glucose standard solution were taken as blank and standard controls. The contents of rapidly digestible starch (RDS), slowly digestible starch (SDS), and resistant starch (RS) in the samples were calculated according to the following Equation (5):

(5) $$\mathrm{RDS}\;(\%)=(G_{20}-\mathrm{FG})\;\times \;0.9\;\mathrm{SDS}\;(\%)=(G_{120}-G_{20})\times 0.9\;\mathrm{RS}\;(\%)=1-(\mathrm{RDS}+\mathrm{SDS})$$

In this formula, G_20_ and G_120_ correspond to the glucose levels in the centrifuge tubes after hydrolysis with the enzyme mixture for 20 min and 120 min, respectively. FG = 0 indicates the glucose level in the centrifuge tubes when no enzyme was added.

#### 2.2.11. Data Analysis

All experiments were replicated at least three times, and the experimental data were analyzed using ANOVA (*p* < 0.05) with IBM SPSS Statistics 26 software. Additionally, the results of water distribution, textural properties, dry matter water absorption rate, cooking loss rate, and in vitro digestion characteristics were analyzed using Origin 2018 software.

## 3. Results and Discussion

### 3.1. Color of Noodles

Color is a crucial indicator in assessing the quality of noodles. It not only forms the first sensory impression that consumers have but also plays a key role in influencing their overall acceptance of the product [29]. The effect of PAW-EYF addition on the variation of color parameters (L*, a*, b*, and WI) of the color bars of noodles is shown in Table 1. Different additions of modified yam flour had a significant effect on the L*, a*, b*, and WI values of the noodles (*p* < 0.05). From Table 1, it can be observed that the brightness of yam noodles decreased with the increase in the PAW-EYF addition, reaching its lowest point (70.50) at a 25% addition. Meanwhile, the corresponding a* value decreased from 5.63 to 3.64, and the b* value decreased from 20.53 to 14.22, indicating that the red and yellow colors of yam noodles were weakened. Additionally, the WI decreased from 71.26 to 67.04, suggesting that the colors of yam noodles gradually darkened. The color change of the yam noodles may have been related to the addition of modified yam flour, where the proteins in the yam flour reacted with sugars through the Maillard reaction or underwent caramelization during high-temperature extrusion, leading to a deepening of the color [30,31]. Additionally, the color of PAW-EYF itself affected the overall color of the noodles, and this color change became more pronounced with an increasing amount of PAW-EYF, as indicated by the significant decrease in the WI of the yam noodles. Existing research has shown that replacing part of the wheat flour with modified yam powder could improve the antioxidant properties of noodles, but further improvements in taste and color were needed to extend their shelf life [32]. Another study found that Chinese yam flour processed via different methods had a significant impact on the acceptability of noodles, especially in terms of color [33]. In conclusion, the addition of modified Chinese yam flour significantly affected the color of noodles, thereby influencing their sensory quality. Different modification methods and addition ratios had different effects on the color and overall acceptability of noodles. Future research can further optimize the modification methods to improve the comprehensive quality of noodles.

### 3.2. Moisture Distribution

**Figure 1:**
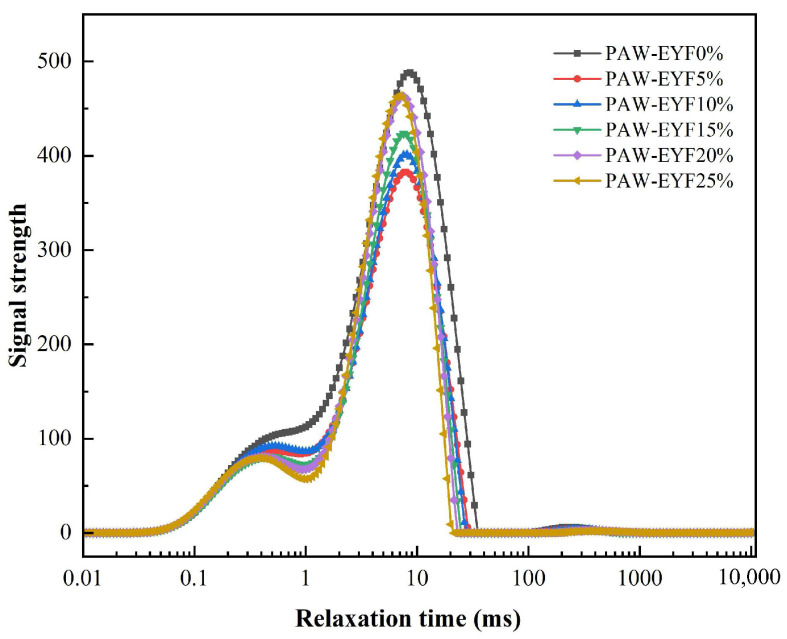
The effect of PAW-EYF addition on the relaxation time of noodles.

Moisture plays a crucial role in the production and processing of noodles. The degree of water binding with starch granules, gluten proteins, and the distribution of water within the noodles significantly affect their quality. Figure 1 shows the variation in the relaxation time of noodles with different additions of PAW-EYF. Each sample curve contained three peaks, representing the different states of water in the noodles: strongly bound water, weakly bound water, and free water, respectively. T_21_, T_22_, and T_23_ denoted the relaxation times of the strongly bound water, weakly bound water, and free water. T_i_ reflected the degree of interaction between water and other components in the noodles. A smaller relaxation time indicated a stronger binding of water, while a larger relaxation time suggested a greater proportion of free water [34]. As shown in Figure 1, compared to wheat noodles, the T_21_, T_22_, and T_23_ values of noodles with different PAW-EYF additions decreased, and the transverse relaxation time shifted to the left. This indicated that the content of bound water increased, the content of free water decreased, and the mobility of water in the noodles weakened.

The moisture distribution of noodles with different amounts of PAW-EYF is shown in Table 1. A_21_, A_22_, and A_23_ represent the ratios of the peak areas of strongly bound water, weakly bound water, and free water, respectively. With the addition of PAW-EYF, the proportions of strongly bound water (A_21_) and weakly bound water (A_22_) increased, while the proportion of free water (A_23_) decreased. This result was consistent with the findings from the transverse relaxation time analysis. This phenomenon may have been attributed to the degradation of yam flour particles following the PAW-TSE treatment, which exposed more hydrogen bonding sites between the starch granules and the protein structures. When PAW-EYF was added to the wheat flour, it enhanced the binding degree between the mixed flour and water, promoting interactions among starch granules and proteins within the dough. This, in turn, strengthened the gluten protein network structure and its ability to bind water, resulting in yam noodles with improved water retention and a more stable structure [35].

### 3.3. Textural Characteristics

**Figure 2:**
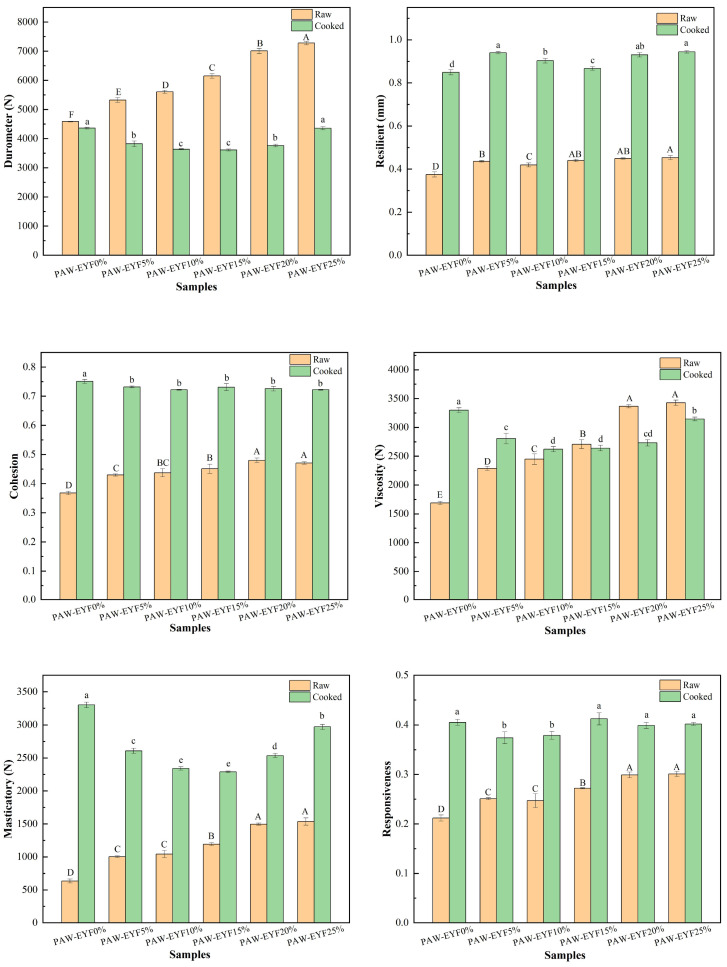
The effect of PAW-EYF addition on the texture properties of raw and cooked noodles. (In the figure, yellow represents the texture profile measurement results for raw noodles, while green represents the results for cooked noodles. Significant differences in the raw noodles are indicated by uppercase letters, whereas significant differences in the cooked noodles are indicated by lowercase letters).

Figure 2 shows the effect of different PAW-EYF additions on the texture of raw and cooked noodles. The measured textural parameters of yam noodles included hardness, elasticity, cohesion, adhesion, chewiness, and reparability. As seen in Figure 2, the hardness of yam noodles gradually increased with the addition of PAW-EYF in the fresh yam noodles. Furthermore, the other textural parameters exhibited a similar increasing trend. The increase in noodle hardness was likely related to decreased water absorption and moisture content, which strengthened the internal structure and resulted in enhanced hardness. The increase in noodle cohesion and chewiness was likely attributed to the pre-gelatinization of PAW-EYF after extrusion, as well as the enhanced internal bonding of the noodles facilitated by the wheat flour during the noodle-making process [36]. For cooked yam noodles, compared with the cooked noodles without modified yam powder, the hardness, adhesiveness, and chewiness of the noodles showed a trend of first decreasing and then increasing. The increase in elasticity and chewiness contributed to a better mouthfeel for the cooked noodles. This might have been related to starch gelatinization, water absorption and expansion, protein denaturation, and changes in the gluten network structure during the cooking process. Overall, yam noodles with a 20% addition of PAW-EYF exhibited the best quality among all the PAW-EYF-added noodles.

### 3.4. Cooking Properties

#### 3.4.1. Optimal Cooking Time and Strip Breakage

Table 2 shows the cooking characteristic parameters of noodles with different PAW-EYF additions. Compared to wheat noodles, yam noodles exhibited shorter cooking times with increasing PAW-EYF additions, decreasing from 2.99 min at 0% to 2.11 min at 25%. This indicated that yam noodles were easier to cook than wheat noodles as the addition increased. Cooking characteristics and breakage rate (i.e., the extent to which noodles break during cooking) are important indicators for assessing noodle quality. These characteristics not only affect the taste and appearance of the noodles but also influence production wastage and consumer satisfaction [37]. As shown in the table, there was no significant difference in the breakage rate of yam noodles. The addition of modified yam flour may have caused changes in the structure of the wheat gluten network, but this change had a minimal effect on the noodle breakage rate. The decrease in the optimal cooking time of the noodles could have been attributed to the fact that PAW-EYF was partially gelatinized during the high-temperature extrusion process. As the addition of PAW-EYF in the yam noodles increased, the content of wheat flour in the noodles decreased, leading to a lower pasting temperature for the mixed powder of PAW-EYF and wheat flour. Additionally, the reduction in the protein content of the mixed powder and the weakening of intermolecular forces may have contributed to the decrease in the time required to cook the noodles.

#### 3.4.2. Absorption of Dry Matter

**Figure 3:**
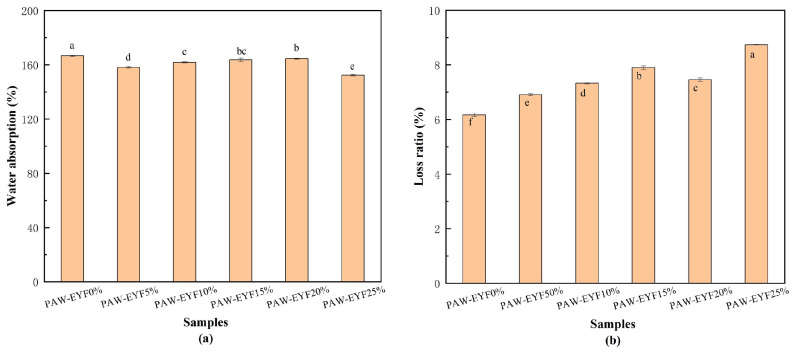
The effects of PAW-EYF addition on the water absorption rate and dry matter loss rate of noodles. (**a**) The effect of PAW-EYF addition on the water absorption rate of noodles, where lowercase letters indicate the significance of differences between treatments. (**b**) The effect of PAW-EYF addition on the dry matter loss rate of noodles, with letters again representing the significance of differences between groups. Identical letters mean no significant difference, while different letters indicate a significant difference.

Dry matter absorbency is defined as the weight of cooked pasta expressed as a percentage of the weight of raw pasta. This measurement represents the ability of the pasta to absorb water during the cooking process [38]. The results of the effect of different PAW-EYF additions on the dry matter water absorption of noodles are shown in Figure 3a. As the PAW-EYF addition increased, the dry matter water absorption rate of yam noodles initially decreased, followed by a slight increase and then a subsequent decrease. This trend can be attributed to the disruption of the powder particle structure of yam flour during the modification process, which reduced water absorption and hindered its ability to closely interact with gluten proteins. Consequently, this led to a significant decrease in the dry matter water absorption of the produced yam noodles. In the noodles with added PAW-EYF, the highest water absorption was observed at a 20% addition, which may have been due to the modified yam flour enhancing the integrity of the gluten network in the dough and encapsulating the yam starch within that network, thus allowing for greater water absorption. Conversely, the lowest water absorption was observed at a 25% addition, which may have been related to the loss of excessive soluble matter during steaming.

#### 3.4.3. Cooking Loss Rate

Cooking loss refers to the degradation of noodle quality resulting from the leaching of substances such as starch granules and small molecules from the gluten network structure during the cooking process [39]. In the noodle production process, a high dry matter water absorption rate, a low cooking loss rate, and a low strip breakage rate are key factors in noodle production, and they indicate the cooking quality of noodles. As shown in Figure 3b, the cooking loss of the noodles increased with the increasing content of PAW-EYF. The use of modified yam starch as a substitute for wheat flour in noodle production, along with yam flour as a gluten-free flour, reduced the gluten content in the mixture. The PAW-EYF reduced the cohesion of the gluten network structure in yam noodles during the cooking process, thereby increasing cooking losses [32]. Additionally, it may also be due to the higher dietary fiber content in yam flour, which can disrupt the formed gluten network, further promoting water penetration into the noodles. The soluble proteins, amylose, and soluble sugars in the noodles may dissolve in water [40].

### 3.5. Microstructure

**Figure 4:**
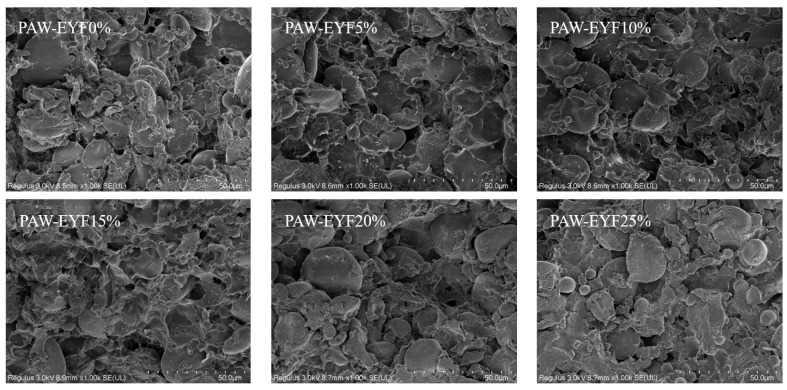
The SEM images of noodles with different additions of PAW-EYF.

In noodles, gluten and starch not only play an important role in supporting the protein network but are also key to maintaining its stability [41]. The SEM microstructures of noodles with different PAW-EYF additions are shown in Figure 4. Compared with wheat noodles, the starch granules in yam noodles were more uniformly and tightly bound to gluten proteins, with aggregation phenomena visible in the cross-section. This phenomenon became increasingly pronounced with the increase in the PAW-EYF addition. This indicates that the addition of modified yam flour increased the binding capacity of starch granules to gluten proteins in the dough, making the starch granules more firmly integrated within the gluten network [38]. In addition, the starch granules on the surface of the noodles were severely deformed when the additive amounts were 5%, 10%, and 15%, and complete starch granules could hardly be seen to be exposed. At 20% and 25%, due to the high addition of PAW-EYF, the degree of starch pasting was reduced, causing changes in shape and preventing fusion between the granules, which allowed clear outlines of the starch granules to be observed.

### 3.6. In Vitro Digestive Properties

**Figure 5:**
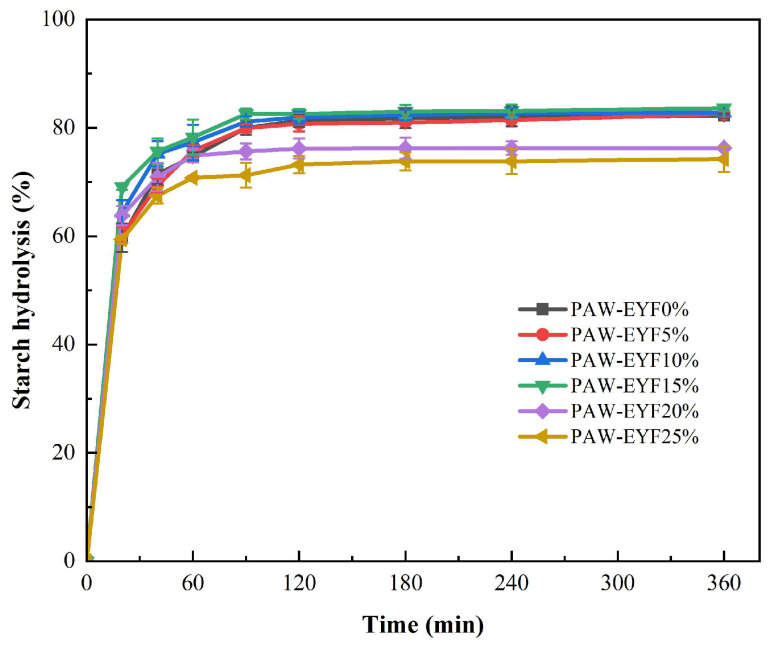
The enzyme hydrolysis curves of noodles with different PAW-EYF addition levels.

**Figure 6:**
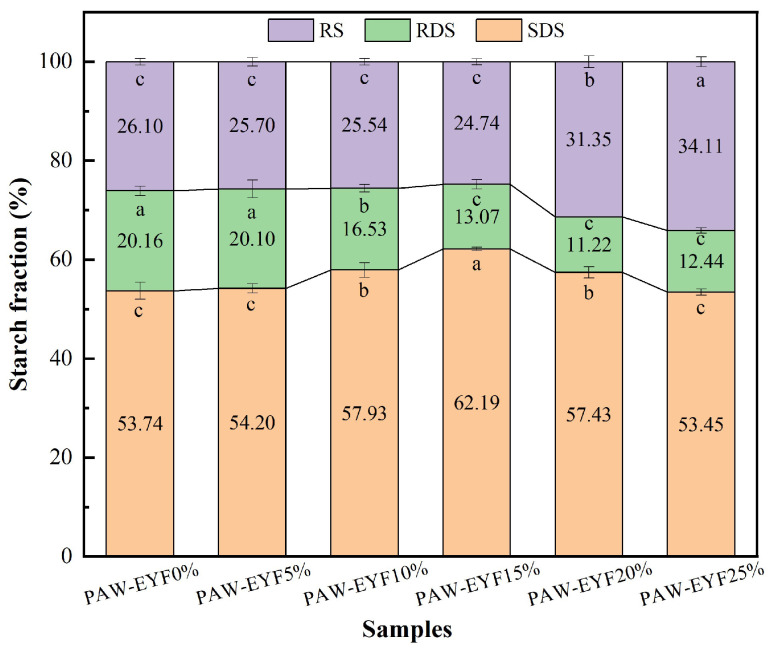
The SDS, RDS, and RS contents of noodles with different PAW-EYF addition levels. The lowercase letters in the figure indicate the significance of differences between groups. Identical letters indicate no significant difference, while different letters indicate a significant difference. It can be observed that the impact of different PAW-EYF addition gradients varies.

Figure 5 shows the enzymatic hydrolysis profiles of noodles with different PAW-EYF additions. The addition of modified yam flour affected the enzymatic hydrolysis rate of starch in the noodles. During the first 20 min of in vitro digestion, the hydrolysis rate of starch increased rapidly, with the highest rate being observed in noodles with a 15% addition. After that, the hydrolysis rate gradually slowed down until it reached equilibrium, and the final hydrolysis rate of noodles with a 25% PAW-EYF addition was the lowest. The contents of SDS, RDS, and RS in noodles with different PAW-EYF additions are shown in Figure 6. As can be seen from Figure 6, the RDS content of yam noodles was reduced compared to that of wheat noodles. With the increase in PAW-EYF addition, the SDS content of the noodles exhibited a trend of increasing and then decreasing, while the RS content showed a trend of decreasing and then increasing. The experimental results are similar to the findings of Ji Xiaolong and others in their previous study [42]. When the additive amount exceeded 20%, the RS content of the noodles was significantly higher, with a maximum of 34.11% being reached. This was mainly due to the higher RS content of PAW-EYF, which was significantly greater than that of wheat flour. The hydrolytic effect of starch digestive enzymes was inhibited as the addition amount gradually increased.

## 4. Conclusions

In this study, modified yam flour (PAW-EYF) was substituted for high-gluten wheat flour to make noodles. The effects of PAW-EYF addition on the color, moisture distribution, textural characteristics, cooking characteristics, microstructure, and in vitro digestive properties of the noodles were investigated. The addition of modified yam flour altered the texture of both raw and cooked noodles, enhancing characteristics such as elasticity and chewiness, which contributed to an improved overall texture in the final product. Furthermore, the modified yam flour increased the resistant starch content of the noodles, suggesting potential health benefits, particularly in terms of digestive health.

The results suggest that yam noodles could provide a healthier and more appealing alternative to traditional wheat-based noodles, aligning with the growing consumer demand for nutritious and functional food options. Future research should focus on further optimizing the production process of yam noodles, investigating long-term storage stability, and exploring broader applications of modified yam flour in various food products. Expanding the use of modified yam flour could play a significant role in the development of healthier, more sustainable food products in the food industry.

## Figures and Tables

**Table 1:** The effect of PAW-EYF addition on noodle color parameters and moisture distribution.

Samples	L*	a*	b*	WI	A_21_ (%)	A_22_ (%)	A_23_ (%)
PAW-EYF 0%	80.70 ± 0.14 a	5.63 ± 0.10 a	20.53 ± 0.13 a	71.26 ± 0.18 a	14.68 ± 0.04 c	84.78 ± 0.02 a	0.54 ± 0.01 a
PAW-EYF 5%	76.91 ± 0.28 b	5.09 ± 0.10 b	17.64 ± 0.16 b	70.50 ± 0.23 b	16.75 ± 0.02 b	82.48 ± 0.03 b,c	0.47 ± 0.02 b
PAW-EYF 10%	74.14 ± 0.49 c	4.86 ± 0.11 c	16.37 ± 0.24 c	69.01 ± 0.32 c	17.25 ± 0.07 a,b	82.52 ± 0.32 b,c	0.44 ± 0.04 b
PAW-EYF 15%	72.20 ± 0.23 d	4.25 ± 0.07 d	15.86 ± 0.20 d	67.71 ± 0.26 d	17.53 ± 0.02 a	82.27 ± 0.24 c	0.38 ± 0.01 c
PAW-EYF 20%	71.34 ± 0.22 e	4.38 ± 0.14 d	15.14 ± 0.23 e	67.29 ± 0.24 d,e	17.50 ± 0.45 a	83.12 ± 0.91 b,c	0.33 ± 0.01 d
PAW-EYF 25%	70.50 ± 0.38 f	3.64 ± 0.08 e	14.22 ± 0.18 f	67.04 ± 0.32 e	16.93 ± 1.03 a,b	83.69 ± 0.79 a,b	0.24 ± 0.00 e

Values are expressed as means ± standard deviations. Different letters in the columns indicate significant differences (*p* < 0.05).

**Table 2:** The effect of PAW-EYF addition on the cooking characteristics of noodles.

Samples	Moisture Content (%)	Optimal Steaming Time (min)	Ratio of Broken Bars (%)
PAW-EYF 0%	25.59 ± 0.26 a	2.99 ± 0.02 a	0.00 ± 0.00
PAW-EYF 5%	24.69 ± 0.91 a,b	2.73 ± 0.01 b	0.00 ± 0.00
PAW-EYF 10%	24.71 ± 0.53 a,b	2.49 ± 0.02 c	0.00 ± 0.00
PAW-EYF 15%	25.50 ± 1.64 a	2.27 ± 0.03 d	0.00 ± 0.00
PAW-EYF 20%	24.03 ± 1.02 a,b	2.16 ± 0.03 e	0.00 ± 0.00
PAW-EYF 25%	23.21 ± 0.71 c	2.11 ± 0.02 e	0.00 ± 0.00

Values are expressed as mean ± standard deviation. Different letters in the columns indicate significant differences (*p* < 0.05).

## Data Availability

The data presented in this study are available upon request from the corresponding author.

## References

[1] Ahmed I., Qazi I.M., Jamal S. (2015). Quality evaluation of noodles prepared from blending of broken rice and wheat flour. Starch-Stärke.

[2] Choo C.L., Aziz N.A.A. (2010). Effects of banana flour and β-glucan on the nutritional and sensory evaluation of noodles. Food Chem..

[3] Hu F., Li J., Zou P., Thakua K., Zhang J., Khan M., Wei Z. (2023). Effects of Lycium barbarum on gluten structure, in vitro starch digestibility, and compound noodle quality. Food Biosci..

[4] Li M., Ma S. (2024). A review of healthy role of dietary fiber in modulating chronic diseases. Food Res. Int..

[5] Gang W., Dan W., Qing C., Chen L., Gao P., Huang M. (2022). Impacts of electron-beam-irradiation on microstructure and physical properties of yam (*Dioscorea opposita Thunb.*) flour. LWT-Food. Sci. Technol..

[6] Yan Y., Xue X., Jin X., Niu B., Chen Z., Ji X., Shi M., He Y. (2022). Effect of annealing using plasma-activated water on the structure and properties of wheat flour. Front. Nutr..

[7] Shen C., Chen W., Aziz T., Al-Asmari F., Alghamdi S., Bayahya S., Cui H., Lin L. (2024). Effects of cold plasma pretreatment before different drying process on the structural and functional properties of starch in Chinese yam. Int. J. Biol. Macromol..

[8] Chaple S., Sarangapani C., Jones J., Carey W., Causeret L., Genson A., Duffy B., Bourke P. (2020). Effect of atmospheric cold plasma on the functional properties of whole wheat (*Triticum aestivum* L.) grain and wheat flour. Innov. Food Sci. Emerg..

[9] Zhao Y., Patange A., Sun D., Tiwari B. (2020). Plasma-activated water: Physicochemical properties, microbial inactivation mechanisms, factors influencing antimicrobial effectiveness, and applications in the food industry. Compr. Rev. Food Sci. Food Saf..

[10] Okyere A.Y., Rajendran S., Annor G.A. (2022). Cold plasma technologies: Their effect on starch properties and industrial scale-up for starch modification. Curr. Res. Food Sci..

[11] Guo D., Liu H., Zhou L., Xie J., He C. (2021). Plasma-activated water production and its application in agriculture. J. Sci. Food Agric..

[12] Jung S., Kim H.J., Park S., Yong H., Choe J., Jeon H., Choe W., Jo C. (2015). The use of atmospheric pressure plasma-treated water as a source of nitrite for emulsion-type sausage. Meat Sci..

[13] Ma R.N., Wang G.M., Tian Y., Wang K., Zhang J., Fang J. (2015). Non-thermal plasma-activated water inactivation of food-borne pathogen on fresh produce. J. Hazard. Mater..

[14] Guo J., Huang K., Wang X., Lyu C., Yang N., Li Y., Wang J. (2017). Inactivation of yeast on grapes by plasma-activated water and its effects on quality attributes. J. Food Protect..

[15] Xu Y.Y., Tian Y., Ma R.N., Liu Q., Zhang J. (2016). Effect of plasma activated water on the postharvest quality of button mushrooms, *Agaricus bisporus*. Food Chem..

[16] Sarangapani C., Scally L., Gulan M., Cullen P. (2020). Dissipation of pesticide residues on grapes and strawberries using plasma-activated water. Food Bioprocess Technol..

[17] Qiao C., Zeng F., Wu N., Tan B. (2021). Functional, physicochemical and structural properties of soluble dietary fiber from rice bran with extrusion cooking treatment. Food Hydrocoll..

[18] Ali S., Singh B., Sharma S. (2020). Effect of processing temperature on morphology, crystallinity, functional properties, and in vitro digestibility of extruded corn and potato starches. J. Food Process. Preserv..

[19] Wang B., Dong Y., Fang Y., Gao W., Kang X., Liu P., Yan S., Cui B., El-Aty A. (2022). Effects of different moisture contents on the structure and properties of corn starch during extrusion. Food Chem..

[20] Yan X., Wu Z., Li M., Yin F., Ren K., Tao H. (2019). The combined effects of extrusion and heat-moisture treatment on the physicochemical properties and digestibility of corn starch. Int. J. Biol. Macromol..

[21] Cheng J., Wang J., Chen F., Wu D., Gao C., Cheng W., Wang Z., Shen X., Tang X. (2023). Effect of low temperature extrusion-modified potato starch addition on properties of whole wheat dough and texture of whole wheat youtiao. Food Chem..

[22] Wani A., Farooq G., Qadir N., Wani T. (2019). Physico-chemical and rheological properties of bengal gram (*Cicer arietinum* L.) starch as affected by high temperature short timeextrusion. Int. J. Biol. Macromol..

[23] Yan Y., Peng B., Niu B., Ji X., He Y., Shi M. (2022). Understanding the structure, thermal, pasting, and rheological properties of potato and pea starches affected by annealing using plasma-activated water. Front. Nutr..

[24] Yan Y., Fang J., Zhu X., Ji X., Shi M., Niu B. (2024). Effect of extrusion using plasma-activated water on the structural, physicochemical, antioxidant and in vitro digestive properties of yam flour. Food Chem..

[25] Zhu F., Cai Y., Sun M., Corke H. (2008). Influence of amaranthus betacyanin pigments on the physical properties and color of wheat flours. J. Agric. Food Chem..

[26] Cankurtaran T.K., Nermin B. (2023). Effect of germinated and heat-moisture treated ancient wheat on some quality attributes and bioactive components of noodles. Food Chem..

[27] Yan Y., Feng L., Shi M., Cui C., Liu Y. (2020). Effect of plasma-activated water on the structure and in vitro digestibility of waxy and normal maize starches during heat-moisture treatment. Food Chem..

[28] Shi M., Song X., Chen J., Ji X., Yan Y. (2024). Effect of Oat Beta-Glucan on Physicochemical Properties and Digestibility of Fava Bean Starch. Foods.

[29] Morris C.F. (2018). Determinants of wheat noodle color. J. Sci. Food Agric..

[30] Ouyang J., Fan K., Li Q., Wang F., Li W., Su X. (2023). Mechanism of feed moisture levels in extrusion treatment to improve the instant properties of Chinese yam (*Dioscorea opposita* Thunb.) flour. Food Chem..

[31] Espinosa-Ramírez J., Rodriguez A., Rosa-Millán J., Heredia-Olea E., Pérez-Carrillo E., Serna-Saldívar S. (2021). Shear-induced enhancement of technofunctional properties of whole grain flours through extrusion. Food Hydrocolloid..

[32] Djeukeu W.A., Gouado I., Leng M.S., Vijaykrishnaraj M., Prabhasankar P. (2017). Effect of dried yam flour (*Dioscorea schimperiana*) on cooking quality, digestibility profile and antioxidant potential of wheat based pasta. J. Food Meas. Charact..

[33] Chin C., Huda N., Yang T. (2012). Incorporation of surimi powder in wet yellow noodles and its effects on the physicochemical and sensory properties. Int. Food Res. J..

[34] Liang J., Maeda T., Tao X., Wu Y., Tang H. (2017). Physicochemical properties of Pueraria root starches and their effect on the improvement of buckwheat noodle quality. Cereal Chem..

[35] Li Y., Liu H., Wang Y., Shabani K., Qin X., Liu X. (2020). Comparison of structural features of reconstituted doughs affected by starches from different cereals and other botanical sources. J. Cereal Sci..

[36] Fu B. (2007). Asian noodles: History, classification, raw materials, and processing. Food Res. Int..

[37] Obadi M., Zhang J., Shi Y., Xu B. (2021). Factors affecting frozen cooked noodle quality: A review. Trends Food Sci. Technol..

[38] Sun K., Liao A., Zhang F., Thakur K., Zhang J., Huang J., Wei Z. (2019). Microstructural, textural, sensory properties and quality of wheat-yam composite flour noodles. Foods.

[39] Guo X., Wei X., Zhu K. (2017). The impact of protein cross-linking induced by alkali on the quality of buckwheat noodles. Food Chem..

[40] Phongthai S., D’Amico S., Schoenlechner R., Homthawornchoo W., Rawdkuen S. (2017). Effects of protein enrichment on the properties of rice flour based gluten-free pasta. LWT.

[41] Niu M., Hou G., Kindelspire J., Krishnan P., Zhao S. (2017). Microstructural, textural, and sensory properties of whole-wheat noodle modified by enzymes and emulsifiers. Food Chem..

[42] Ji X., Chen J., Jin X., Chen J., Ding Y., Shi M., Guo X., Yan Y. (2023). Effect of Inulin on Thermal Properties, Pasting, Rheology, and In Vitro Digestion of Potato Starch. Starch-Stärke.

